# Epicardial Contribution to the Developing and Injured Heart: Exploring the Cellular Composition of the Epicardium

**DOI:** 10.3389/fcvm.2021.750243

**Published:** 2021-09-23

**Authors:** Thomas J. Streef, Anke M. Smits

**Affiliations:** Department of Cell and Chemical Biology, Leiden University Medical Center, Leiden, Netherlands

**Keywords:** epicardium, heterogeneity, development, cardiac repair, single-cell RNA sequencing

## Abstract

The epicardium is an essential cell population during cardiac development. It contributes different cell types to the developing heart through epithelial-to-mesenchymal transition (EMT) and it secretes paracrine factors that support cardiac tissue formation. In the adult heart the epicardium is a quiescent layer of cells which can be reactivated upon ischemic injury, initiating an embryonic-like response in the epicardium that contributes to post-injury repair processes. Therefore, the epicardial layer is considered an interesting target population to stimulate endogenous repair mechanisms. To date it is still not clear whether there are distinct cell populations in the epicardium that contribute to specific lineages or aid in cardiac repair, or that the epicardium functions as a whole. To address this putative heterogeneity, novel techniques such as single cell RNA sequencing (scRNA seq) are being applied. In this review, we summarize the role of the epicardium during development and after injury and provide an overview of the most recent insights into the cellular composition and diversity of the epicardium.

## Introduction

Ischemic heart disease, and especially myocardial infarction (MI) remains a major cause of death globally (1). MI is primarily caused by obstruction of the coronary vasculature, and the resulting sudden loss of oxygen supply to the cardiac muscle leads to massive cell death. Cardiomyocytes lack the ability to sufficiently self-renew and therefore they are unable to replenish the lost muscle. Instead, dead cells are replaced by a fibrotic scar (2, 3). While this non-contractile scar protects the damaged myocardial wall from rupture, it also impairs proper cardiac contraction. This persistent loss of cardiac pump function eventually results in heart failure (HF), a disease for which a cardiac transplant is the only curative therapy. Since intrinsic repair mechanisms are insufficient to restore cardiac function after injury, the focus shifted to inducing cardiac repair through other means. These procedures include the direct injection of various (stem) cell populations to generate new tissue, or the delivery of exosomes or paracrine factors to induce vascularization and prevent apoptosis. Many of these approaches resulted in some degree of improved heart function after MI in pre-clinical studies (4–7). However, the anticipated promise of cell-therapy was not upheld after transition to clinical trials: the results of cell injections on cardiac function in patients have been inconclusive (8). This leaves the mechanisms underlying the observed positive effect in pre-clinical studies unclear (4), but it demonstrates that cardiac regeneration requires more than solely the injection of cells. Another approach that is currently under investigation to achieve cardiac regeneration is through the stimulation of endogenous cell populations that eventually replace the lost cells and stimulate repair. This includes stimulating the local endothelium to increase vascularization (9), as well as the option to induce proliferation in pre-existing cardiomyocytes to create new contractile units (10–12). In this regard, an interesting candidate for endogenous repair that has seen increasing attention is the epicardium.

### The Epicardium as an Endogenous Cell Population for Cardiac Repair

The epicardium is a single-cell layer of mesothelial origin located on the outside of the heart. Intriguingly, this cell type is of crucial importance during cardiac development. In brief, the epicardium contributes cardiac cell types to the developing heart (13, 14), it facilitates the formation of the coronary vasculature (15, 16), it can induce the proliferation of cardiomyocytes through the secretion of paracrine factors (17–19), and derivatives of the epicardium can modulate the extracellular matrix (20). All these processes are also essential to repair the heart after injury.

In the healthy adult heart, the epicardium is a quiescent layer. However, it is reactivated after certain types of injury and subsequently it recapitulates several of its developmental processes. In animal models that display the potential for cardiac regeneration such as zebrafish and neonatal mammals (21, 22), the epicardium has been shown to play an important role in facilitating and regulating processes involved in repair, including modulation of inflammatory responses and of the composition of the extracellular matrix, secreting paracrine factors, and contributing cells to the damaged heart (23–25). These observations prompted researchers to attempt to stimulate the adult epicardium in mammals to increase its participation in repair (26).

To optimize the post-injury response, it is important to understand the processes underlying the activation of the epicardium and the regulation of its differentiation into cardiac cell types. An unresolved question in this context has been whether the whole epicardial population can participate, or whether distinct cell types reside within the epicardial layer that have specific abilities within the reparative response. With the advent of single cell sequencing, we are gaining more insight into the composition and the potential contribution of endogenous cells in the heart. Here, we will highlight the role of the epicardium during development and cardiac repair and discuss novel insights on the composition of this cell layer based on single cell RNA sequencing (scRNA seq) data.

## The Epicardium in Heart Development

### The Proepicardium and the Formation of the Epicardium

As stated above, the epicardium has an important function in the formation of the heart during embryogenesis. The developmental origin of the epicardium lies within the proepicardial organ (PEO). The PEO is an evolutionary conserved cluster of cells that develops from the lateral plate mesoderm and is located at the venous pole of the heart near the septum transversum. In mice, the PEO becomes visible around embryonic day 8.5 (E8.5) (27), a stage when the developing heart is still a primitive tube-like structure. After E9.5, when the heart tube has started to loop and form distinguishable segments such as the primitive left ventricle and outflow tract, cells from the PEO start to translocate and attach to the outside of myocardium, where they will ultimately form the epicardium. In mammalian development this proepicardial translocation has been described to occur *via* the formation of free-floating cell aggregates or *via* direct contact with the myocardium (28–30), while in avian and zebrafish models cells from the PEO are likely to migrate toward the heart *via* a “bridge” consisting of extracellular matrix components such as heparan sulfate and fibronectin (31, 32). Upon reaching the bare myocardium, cells from the PEO flatten and form a continuous epithelial layer that will completely cover the heart around E12.5 in mice and week 5 in human cardiac development (33, 34).

Identification of the PEO as a transient structure has relied on scanning electron microscopy (SEM) (27) and staining with specific antibodies. The most commonly used markers to identify the PEO include for example transcription factor 21 (TCF21), T-box transcription factor 18 (Tbx18), Wilms' Tumor-1 (Wt1), Scleraxis (Scx), Semaphorin3D (SEMA3D), and GATA5 (31, 35–39). The expression of some of these markers persists after the epicardium is formed and they are therefore often also used to identify the epicardial layer in later developmental stages. But as will become clear, these markers have a heterogeneous spatiotemporal expression in the PEO and in the epicardium throughout development. This could suggest the existence of subtypes of cells that have distinct roles in cardiogenesis, or even in regeneration of the injured heart.

### Cellular Contributions of the Epicardium During Development

The vital role of the epicardium for cardiac development was highlighted by studies in an avian model where epicardial outgrowth from the PEO was physically inhibited. This led to the formation of a thin myocardium and malformation of the coronary vasculature, amongst other developmental defects (13, 40), indicating that the epicardium is more than a static epithelial cell layer enveloping the heart. Indeed, once the epicardium is fully formed a subset of the epicardial cells will undergo a process called epithelial-to-mesenchymal transition (EMT), thereby forming epicardium-derived cells (EPDCs) (41, 42). EMT is a well-described process which is crucial in embryonic development but also observed in diseases such as metastatic cancer and fibrosis (43). During EMT, epithelial cells lose their apical-basal polarity and cell-cell adhesions, and acquire a mesenchymal phenotype that allows the migration and invasion of cells into tissue (44). EMT-derived mesenchymal cells have the potential to differentiate into various mesenchymal cell lineages, such as adipocytes, chondrocytes, and osteoblasts (45). A similar feature is observed in cells derived from the epicardium; EPDCs have been reported to differentiate into various cell types, including fibroblasts, pericytes and smooth muscle cells (SMCs) (14, 46–49). Other reports claim that the epicardium upon EMT also contributes cells to endothelial cell (EC) lineages and to the cardiomyocyte (CM) population. However, these findings are under debate and an epicardial contribution to these tissues is likely very limited at best (49–52).

**Figure 1:**
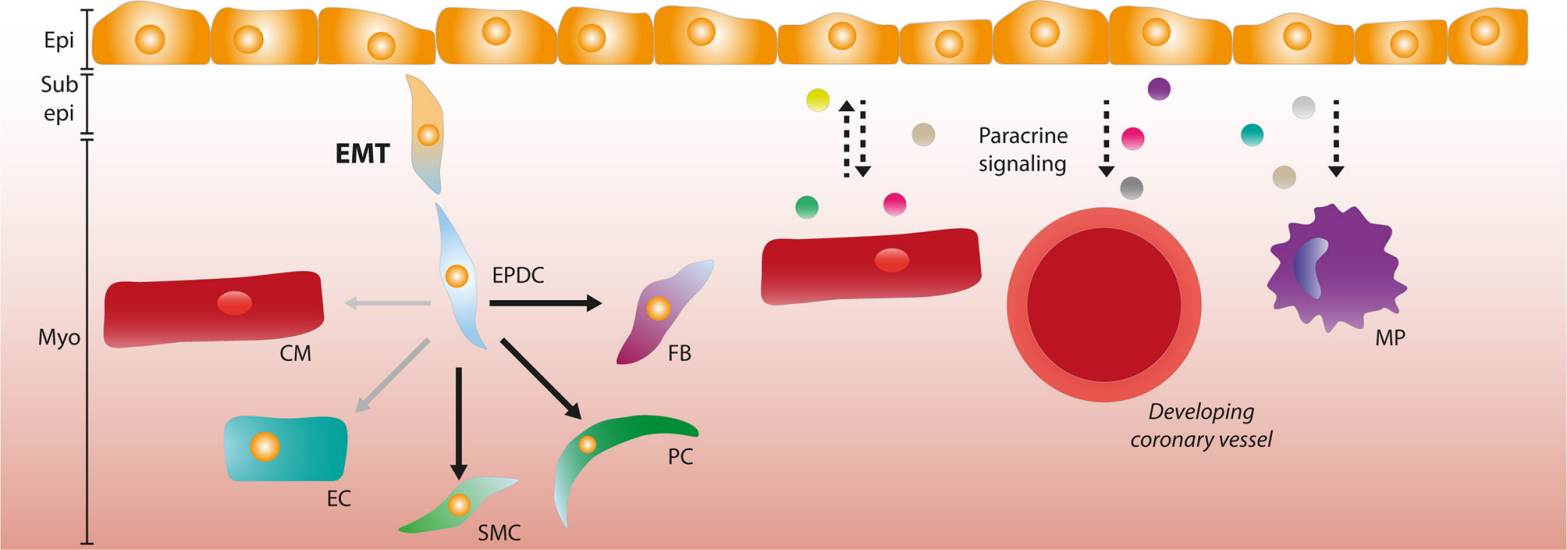
The role of the epicardium during development. Epicardial cells can undergo epithelial-to-mesenchymal transition (EMT) and form epicardium-derived cells (EPDCs) that migrate through the sub-epicardial space into the myocardium. EPDCs can differentiate into various cardiac cell types such as fibroblasts (FBs), pericytes (PCs), and smooth muscle cells (SMCs). The contribution to the endothelial cell (EC) lineage is limited (as depicted by opaque and smaller arrow), and the capacity to differentiate into cardiomyocytes (CMs) is debated. Paracrine signaling interactions (depicted by dashed arrows) occur between the epicardial layer and CMs, and epicardial signaling is involved in coronary vessel formation and macrophage (MP) recruitment. Epi, Epicardium; Sub epi, Subepicardium; Myo, Myocardium.

A possible explanation for these discrepancies in differentiation capacity is because analysis of cell fate is mainly based on lineage-trace models where Cre-recombinase (Cre) is driven by promoters that are considered specific to epicardial cells. By crossing these mice with transgenic reporter lines containing a lox-flanked stop-codon followed by a reporter gene, cell specific Cre expression results in indefinite expression of a reporter protein like Green Fluorescent Protein (GFP) or β-galactosidase. Several promoters of epicardial related genes such as *Wt1, Tbx18, Tcf21, GATA5, Scx*, and *Sema3D* have been used to trace the fate of epicardial cells based on transgene expression. An even better controlled lineage trace system can be achieved by fusing Cre to a mutated ligand-binding domain of the human estrogen receptor, in which recombination relies on the presence of tamoxifen. This provides lineage tracing with a temporal control (53), as demonstrated in mice by using promoters of *Tbx18, Wt1*, and *Tcf21* in zebrafish (37, 54, 55). Unfortunately, most of the promoters used in epicardial lineage-tracing models are not uniformly expressed in the epicardium and have a dynamic temporal expression pattern in the epicardium and its derivatives. Additionally, they can also be expressed in the PEO, and in various other cell types of the developing heart, such as ECs and CMs (31, 56–58). As a result, lineage trace models can potentially label cells that not necessarily originate from the epicardium. This problem was highlighted by a study comparing various lineage-tracing models and the contribution of different lineages to the EC population in the heart. Carmona et al. showed that Wt1 lineage-trace models should not be used after E13.5, since *de novo* expression of Wt1 in other tissues (i.e., endothelium) arises as well as through recruitment of extracardiac progenitors (51). However, the authors found that EPDCs contribute roughly 4% of the coronary endothelium using GATA5^Cre^ mice (51). Others reported that coronary endothelium expressed Wt1 as early as E11.5 in a Wt1^CreERT2^ model, meaning that tamoxifen should be administered at E9.5 to prevent labeling of coronary ECs (59). Interestingly, it has been argued that since epicardial markers *Wt1, Sema3d, Tbx18, Scx*, and *Tcf21* overlap, and Tbx18^Cre^ and Tcf21^CreERT2^ show no endothelial contribution, that this applies to the entire (pro)epicardium (59). Nevertheless, carefully controlled lineage-trace models have still provided valuable insight into the cell fate of EPDCs and the mechanisms steering epicardial differentiation, and the current consensus is that EPDCs have the capacity to differentiate into SMCs, fibroblasts and pericytes, and potentially ECs (Figure 1). Additionally, these models have shown that the epicardial EMT is crucial for cardiac development.

### Regulation of Epicardial EMT and Differentiation

Epicardial EMT is a defining process for the contribution of epicardial cells to tissue formation as it grants cells the capacity to migrate and differentiate. In general, epithelial cells preserve their phenotype *via* expression of epithelial cadherin (CDH1, E-Cadherin), which is responsible for maintaining cell-cell adhesion and adherens junctions. The EMT-inducing transcription factors, including Snail Family Transcriptional Repressors 1 and 2 (SNAI1/2), zinc finger E-box binding family members 1 and 2 (ZEB1/2), and twist-related protein 1 (TWIST1) (44) can all repress CDH1 and simultaneously activate the expression of mesenchymal genes. Interestingly, the most commonly used markers to identify the epicardium like Wt1, Tcf21, and Tbx18 also appear to have a role in the regulation of EMT upstream of these factors (60). Wt1 is a zinc-finger protein, initially recognized for its role in the formation of Wilms' tumor, that was found to be expressed in both the PEO and the epicardium, and during EMT (33, 61, 62). Wt1 regulates epicardial EMT through transcriptional activation of Snai1 as well as a direct repression of E-cadherin (63, 64). However, in mouse embryos the removal of *Snai1* specifically in Wt1- or Tbx18-positive epicardial cells did not affect cardiogenesis, and embryos displayed normal epicardial EMT (65). This suggests that SNAI1 may not be the sole inducer of EMT, and that compensatory mechanisms are in place. In contrast, embryos that lack *Wt1* were found to have severe epicardial defects with an absence of EPDCs in the subepicardial mesenchyme and impaired cardiac morphogenesis, resulting in embryonic lethality at E13.5 due to pericardial bleeding (16, 62). Additionally, *Wt1* knockout mice revealed a role for Wnt/β-catenin and retinoic acid signaling pathways downstream of WT1 (16). Indeed, in additional studies epicardial β-catenin signaling was found to be crucial for epicardial EMT, myocardial invasion, and differentiation into coronary smooth muscle of EPDCs (66).

Tbx18 is a marker commonly used to identify the epicardium and that is also expressed in the PEO (36). *In vitro*, using mouse primary epicardial cells, a bi-directional role for WT1 and Tbx18 was reported. *Wt1* knockdown induced epicardial EMT through expression of *Snai2*, which could be reversed by knockdown of Tbx18, thus acting as a regulator of EMT (67). *Tbx18*- lineage-tracing models have shown that *Tbx18*-positive cells differentiate into SMCs and fibroblasts (46), and this was corroborated by a study in which an activating form of Tbx18 induced EPDCs to undergo a pre-mature differentiation into SMCs mediated by Notch and transforming growth factor β (TGFβ) (68). In contrast, *Tbx18* null embryos survive until birth and die due to skeletal malformations, indicating that Tbx18 is dispensable for epicardial development (68).

Tcf21 (also known as Pod1/epicardin/capsulin), a class II basic helix-loop-helix (bHLH) transcription factor, is another epicardially expressed protein that is also involved in the regulation of EMT and the differentiation into various cellular lineages. Depletion of Tcf21 during early stages in *Xenopus* development led to incomplete formation of a mature epithelial epicardium. Additionally, Tcf21 depletion resulted in the epicardial cells retaining a migratory phenotype, as they maintained their PEO cell-like phenotype (69). In *Tcf21* null mice, epicardial cells lacked the ability to become mesenchymal cells, indicating that Tcf21 is required for EMT. In the same study, it was shown that Tcf21^+^ cells were committed to a cardiac fibroblast fate, supporting the importance of Tcf21 in epicardial differentiation (48). The regulation and downstream effects of Tcf21 may be more intricate, as another study showed that Tcf21 was regulated by retinoic acid signaling and that absence of Tcf21 led to an increased smooth muscle cell differentiation, but that EMT was unaffected (57). These studies showed a complex regulatory role for Tcf21 in differentiation of EPDCs, and further investigation into these cell fate decisions is needed to elucidate the mechanisms behind it.

Besides the “classical” markers of the epicardium, other epicardial transcription factors have also been described to regulate epicardial characteristics beyond EMT, like migration and invasion into the myocardium. These include for instance the myocardin-related transcription factors (MRTFs), nuclear factor of activated T-cells 1 (NFATC1) and protein arginine methyltransferase 1 (PRMT1). The MRTF serum-response factor (SRF) regulatory network modulates epicardial migration and invasion. Cell motility is driven by interactions between SRF and MRTF-A/B, modulating the expression of regulators of actin dynamics (70). In an *ex vivo* heart culture model, deletion of *Mrtfa* and *Mrtfb* reduced EPDC migration. Additionally, in embryos lacking both *Mrtfa* and *Mrtfb*, epicardial integrity was compromised, as well as the coronary angiogenesis due to reduced epicardium-derived pericytes (71). NFATC1, a transcription factor involved in extracellular matrix (ECM) remodeling during valve maturation (72), is expressed in a subset of epicardial cells within the epicardial layer and in EPDCs in the subepicardial space (73). It was found to influence the invasion of EPDCs into the myocardium through enhancing cathepsin K expression, an ECM degrading enzyme. *Wt1-Cre* mediated deletion of NFATC1 in mice led to a reduction in the number of α-smooth muscle actin-expressing EPDCs in the myocardium, as well as a reduced intramyocardiac vessel penetration and fibrous matrix synthesis (73). However, in this model, the initial stages of epicardial formation and EMT were not affected, indicating that modulation of the ECM required for EPDC invasion is affected in part by NFATC1.

Molecular regulation of epicardial behavior beyond transcription factors also occurs. A newly identified regulator of epicardial EMT is PMRT1, an arginine methyltransferase responsible for post-translational modifications. PRMT1 knockout mice showed a reduced migration of EPDCs and an attenuated formation of EPDC-derived lineages such as fibroblasts, SMCs, and pericytes. The mechanism of these processes is likely the stabilization of p53 due to the loss of PRMT1, and higher levels of p53 lowered the expression SNAI2, and thereby blocked epicardial EMT, confirming a role for *Snai2* in epicardial EMT in mice. Interestingly, the reduction of p53 levels in Tbx18-mediated PRMT1 knockout mice normalized the disrupted invasion, as well as the formation of epicardium-derived mesenchymal lineages (74). In short, the epicardial contribution to various cell lineages is of great importance for proper development of the heart. The genes that are involves in these processes are in several cases also used to identify the epicardium, although their expression may not be uniform due to spatiotemporal control.

### Paracrine Signaling During Development

Besides a cellular contribution, the epicardium is a rich source of growth factors and cytokines and as such it provides essential cues for cardiac development including factors that support cardiomyocyte proliferation and vessel formation. The epicardium expresses various members of the fibroblast growth factor (FGF) family and its receptors (FGFR). For example, FGFR1 is expressed both in the PEO and in the epicardium, and loss of FGFR1 in quail embryos was shown to reduce the myocardial invasion of epicardial cells (75). In contrast, in a *Tbx18*^*Cre*^ mediated deletion of *Fgfr1* and *Fgfr2* in mice no differences in myocardial fibroblast numbers were observed, and importantly cardiac development was not affected (76), indicating that in Tbx18^+^ cells FGFR signaling is not required for fibroblast invasion into the myocardium.

FGF9 is another family member related to the epicardium. Epicardial FGF9 is induced by retinoic acid (RA) produced by the epicardium and promotes proliferation and differentiation of cardiomyocytes *via* receptor splice variants FGFR1c and FGFR2c (17). Additionally, FGF9 signaling plays a role in the formation of the coronary vasculature (77, 78). Besides inducing expression of FGF9, RA signaling leads to expression of *Wt1* and *Tcf21* (57), two TFs that regulate epicardial EMT, and it stimulates myocardial expansion *via* IGF2 (79). RA also has an epicardium-specific role, since epicardial specific knockout of RA receptor Retinoid X Receptor a (RXRa) mediated by *Gata5*^*Cre*^ led to reduced EMT, cardiac compaction, and defects in coronary arteriogenesis *via* impaired FGF2 signaling (80). Conversely, signaling from the myocardium to the epicardium also occurs. Myocardial signaling to the epicardium is for instance mediated by FGF10. FGF10 is expressed by cardiomyocytes during development and it stimulates invasion of EPDCs into the myocardium, and their differentiation to fibroblasts *via* FGFR2b (81).

The epicardium also interacts with the developing coronary vasculature. It was found that *Wt1*-KO mice have deficient epicardial expression of angiogenic factors *Vegfa* and *Angpt1*, suggesting a contribution to abnormal coronary vessel development (16). C-X-C motif chemokine 12 (CXCL12) is expressed by the epicardium and by mesenchymal cells derived from the epicardium, it was shown to be crucial for the maturation of the coronary vasculature *via* C-X-C motif receptor 4 (CXCR4) on nearby endothelial cells (82). A single factor was found using single-cell sequencing of developing mouse hearts at E10.5. The authors found that *Rspo1* is expressed by epicardial cells and which was hypothesized to promote proliferation of compact myocardium (83). Another important epicardial signaling family comprises the platelet-derived growth factors (PDGFs). PDGFA and PDGFB are both expressed by various cardiac cell types, including the epicardium, and mediate various aspects of cardiac development (84–87). Its receptors, PDGFRα and β, are both expressed in the epicardium (84, 88), and loss of these receptors led to defective epicardial EMT and migration *in vivo. Ex vivo* these hearts displayed decreased epicardial migration also in the presence of EMT-inducing growth factors TGFβ1 and FGF2 (89). *In vitro*, expression of *Sox9* in PDGFR-deficient epicardial cells partially rescued the deficient EMT, implicating a signaling pathway downstream of PDGFR-signaling regulating this transcription factor (89). Moreover, epicardial loss of PDGFRα and PDGFRβ resulted in a reduction in myocardial fibroblasts and SMCs, respectively, indicating that these receptors likely play a role in epicardial cell differentiation and migration (76, 88).

Besides signaling to and from other cardiac cell types, the epicardium also secretes factors that can function in an autocrine fashion. TGFβ is a well-established inducer of EMT, and its isoforms are present during (pro)epicardial development (90, 91). TGFβ1 and TGFβ2 induce loss of epithelial morphology and the differentiation into SMCs through ALK5 signaling, the TGFβ type I receptor (92). Concordantly, a *Gata5* mediated *Alk5* knockout prevented EMT upon TGFβ3 stimulation and reduced the number of proliferating cardiomyocytes. Furthermore, it impaired adherence of the epicardial layer to the myocardium, and diminished differentiation into SMCs due to a lack of epicardial EMT (93). Moreover, mice embryos lacking ß-glycan, also known as *Tgfbr3*, have a diminished coronary vessel development and hyperplasia of the subepicardial layer due to decreased proliferation and invasion of EPDCs (94–97). Overall, these studies highlight the importance and complexity of the regulation of paracrine signaling in epicardial and cardiac development (Figure 1).

## The Adult Epicardium

### Cellular Contributions From the Adult Epicardium

In contrast to the developing epicardium, in the adult heart the epicardium displays limited Wt1 expression and under homeostatic conditions it does not actively contribute cells to the myocardium (98). Other genes that are expressed in embryonic, active epicardium, such as *Tbx18* and *Raldh2*, are merely expressed at low levels, indicating that the epicardium is in an inactive state in the healthy adult heart (99, 100). However, after ischemic insults like MI the epicardium covering the injured area is lost and the remaining epicardium will start to proliferate and migrate to re-cover the heart (100). This wound healing process leads to a thickening of the epicardial layer near the site of injury, instead of a single-cell layer in a normal heart (98, 100, 101). This reactivation seems to be specific to ischemic injury and is not observed in cardiac hypertrophy models such as transverse aortic constriction (102). Importantly, after ischemic injury, the expression of the epicardial genes *Wt1, Tbx18, Raldh1*, and *Raldh2* is reactivated, peaking 3 days after injury and subsiding after 2 weeks (100). Interestingly, this re-expression occurs throughout the entire epicardium and is not restricted to the site of injury (98). *Wt1* was found to be induced by hypoxia-inducible factor 1a (HIF1a) in ECs (103), which could explain its reactivation after cardiac ischemia, but not after pressure-overload. Furthermore, HIF1a regulates epicardial invasion during development (104), indicating that HIF1a could be a central regulator of the epicardial post-injury response.

Much effort has been put into identifying the regulatory elements that activate the epicardium after injury. In the embryonic heart and after injury, the CCAAT/enhancer binding protein (C/EBP) family of transcription factors was identified as a regulator of *Wt1* and *Raldh2* by binding to their enhancer elements (105). More recently, the transcription activator BRG1 was found to be recruited to conserved regulatory elements in the *Wt1* locus by C/EBPβ and thereby induced *Wt1* expression (106).

Based on knowledge gained from cardiac development, it was anticipated that autonomic recapitulation of an embryonic gene program would result in epicardial EMT and subsequently to a contribution of the adult epicardial derived cells to various cardiac cell types after injury. Several groups have addressed this using lineage tracing, but the results have varied based on the mouse model that was used (107). When using a Bacterial Artificial Chromosome (BAC)-Wt1^Cre^ lineage-trace model, cells derived from the epicardium were reported to contribute to fibroblast, EC and CM lineages (100). Moreover, using a Wt1^Cre^/R26R^LacZ^ lineage-tracing model, Duan et al. reported that cells expressing *Wt1* adopt a fibroblast fate, but other differentiation trajectories were not investigated (101). However, in a similar study using Wt1^CreERT2/+^;Rosa26^mTmG/+^ mice, the differentiation into EC and CM was not observed after MI, decreasing the likelihood of these differentiation trajectories to occur (98). Indeed, as shown by various lineage tracing experiments, the contribution of the epicardium to EC and CM is likely very limited at best after MI (108, 109), and the current conception is that ECs and CMs that arise after injury derive from resident populations in the heart (110–112). Although the multipotency of epicardial cells during development is still under debate, it was established that after injury there appears to be a limited multipotency of epicardial cells in the postnatal heart (113). It is important to note that in the adult, epicardial markers often used in lineage tracing experiments, similar to developmental studies, are not specific enough to label the entire epicardium and cells derived from the epicardium could be missed (114). Other approaches to labeling EPDCs such as MRI-based molecular imaging are being developed, but their specificity *in vivo* has not been determined yet (115, 116).

Although the intrinsic cellular contribution of adult epicardium after injury may be limited, migration of the re-activated epicardial cells appears to be a part of the epicardial injury response. Epicardial reactivation after injury has mostly been shown to have a beneficial effect on cardiac function after MI (98, 99, 101). Therefore, considering the marginal cellular contribution to the injured heart it could be relevant to promote epicardial proliferation and migration *via* external stimuli. One approach to stimulate cardiac repair *via* the epicardium is by treating mice with thymosin ß4 (Tß4), a peptide secreted by endothelial cells and the epicardium during development and after injury (117–119). Importantly, treatment post-MI resulted in an increase in proliferating EPDCs and neovascularization of the injured heart (120). Somatic and cardiomyocyte- and endothelium-specific knockout of Tß4 did not lead to impaired cardiac development or function (121, 122), while shRNA knockdown of Tß4 in CMs and ECs resulted in cardiac defects (123, 124). The discrepancy between these two models could be due to genetic compensatory mechanisms in complete knockouts, while shRNA induced knockdown does not induce a similar compensation (125). Although the mechanism is incompletely understood, a likely explanation is that Tß4 interacts with BRG1, a transcriptional regulator of *Wt1* expression, and that exposure to Tß4 before injury increased the expression of *Wt1* (106). In line with these findings, systemic Tß4 injections prior to injury in the adult mouse resulted in a recapitulation of an embryonic gene program in both healthy and injured hearts and differentiation into cardiomyocytes (99). However, when Tß4 was given post-injury, differentiation of EPDCs into cardiomyocytes was not found (108).

In parallel to the developing heart, several studies imply that besides a cellular contribution there is an important role for paracrine factors secreted by the activated epicardium. These paracrine factors can be used to increase the regenerative potential of the epicardium and of the heart.

### Paracrine Signaling After Injury

The reactivation of the epicardium coincides with the secretion of paracrine factors that can contribute to cardiac repair (98). In a study using lineage tracing in EPDCs in Wt1^CreERT2/+^;Rosa26^mTmG/+^ mice after MI, the authors observed a higher localization of vessels near the GFP^+^ cells. Using fluorescence activated cell sorting (FACS) to isolate these cells, they found that the EPDCs secrete pro-angiogenic factors *in vitro*. Further analysis revealed that FGF2 and VEGFA were in large part responsible for these effects (98). Injection of EPDC conditioned medium after MI increased vessel density and reduced adverse remodeling in both the long and short term. Treatment with a single injection of conditioned medium immediately post-MI also displayed a beneficial effect on cardiac function 1 week after injury, although this effect was not sustained after 9 weeks (98).

In a study in adult zebrafish using cardiac cryoinjury, epicardial Cxcl12b-Cxcr4a signaling was found to guide coronary revascularization. Moreover, the expression of Cxcr12b was induced by hypoxia through Hif1a, again underlining the importance of this factor in regulating the cellular response to ischemic injury (126). Interestingly, these newly formed coronary vessels also functioned as a scaffold for regenerating cardiomyocytes, indicating a new function for the vasculature besides facilitating exchange of nutrients and oxygen. A comparable paracrine pro-angiogenic effect was observed in MI hearts that were treated by transplanting human EPDCs into the borderzone of the injury. Since the injected EPDCs were not found in the vessel lining while there was an increase in vessel density throughout the entire left ventricle regardless of number of engrafted EPDCs, this pointed to a predominantly paracrine effect of the injected cells (127). Human adult EPDCs were also shown to stimulate neurite outgrowth *in vitro* (128), indicating that EPDCs could have an effect on multiple cell types after injury.

Besides a mixture of secreted factors by the epicardial cell layer after injury, single components identified in the epicardium can also be used to enhance cardiac repair. For instance, Follistatin-like 1 (FSTL1), a factor present within the secretome of adult rat epicardial cells *in vitro*, was found to induce cardiomyocyte proliferation *in vivo* when locally applied onto the infarcted area in mouse and swine (129). Interestingly, when investigating the endogenous *in vivo* expression of FSTL1, it was apparent in the epicardium during development and in the adult, but after MI the expression of FSTL1 shifts to the myocardium (129). This finding was confirmed in another study, where the authors established that FSTL1 expression after MI is localized to activated cardiac fibroblasts (110). Nevertheless, increasing local levels of FSTL1 may provide a way to positively affect cardiac function. In a study where modified RNA coding for VEGF-A, a paracrine factor also produced by EPDCs (98), was injected into the infarct zone of the myocardium, an increase in proliferating epicardial cells was observed. Also, an improved migration of EPDCs into the myocardium, and a contribution to EC and SMC populations (130). Similarly, upon the injection of brain natriuretic peptide (BNP) post-MI an increase in proliferation and migration of Wt1-positive cells was observed in Wt1^CreERT2^;Rosa26^mTmG^ mice, together with an increased contribution to the EC lineage (131). In both studies, the contribution to the EC population should be carefully interpreted due to native expression of Wt1 in endothelial cells after injury, which could lead to labeling of ECs in lineage-tracing experiments (114). In a mouse neonatal heart regeneration model epicardial cells were shown to secrete RSPO1, a factor that promotes angiogenesis *in vitro*, suggesting that this factor can promote revascularization after injury (132). Moreover, as this factor was shown to induce cardiomyocyte proliferation in the developing heart (83), its expression after injury may even have more potential to influence the regenerative response after injury.

### Modulation of the Extracellular Matrix by the Adult Epicardium

**Figure 2:**
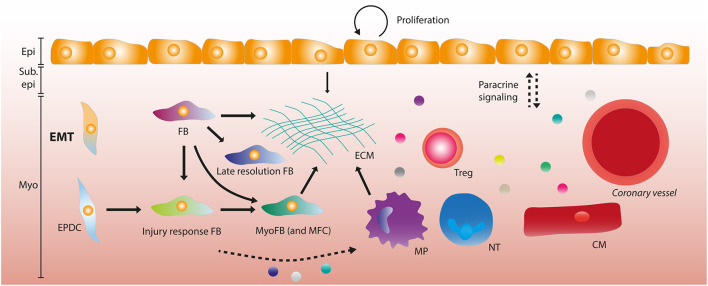
The adult epicardium and stromal heterogeneity after myocardial infarction. After myocardial infarction (MI), the epicardial layer starts proliferating in order to regenerate the lost cells. A subset of epicardial cells undergo epithelial-to-mesenchymal transition (EMT) forming epicardium-derived cells (EPDCs). EPDCs and resident fibroblasts (FBs) can form various stromal cell subtypes, being late resolution FBs and injury response FBs that differentiate into myofibroblasts (MyoFBs) and matrifibocytes (MFCs). Several of these FB subgroups can contribute to extracellular matrix (ECM) deposition. Macrophages (MPs) and the epicardial layer also contribute to ECM formation. Paracrine signaling (depicted by dashed arrows) occurs between the epicardial layer and leukocytes (MPs, neutrophils (NTs) and regulatory T cells (Tregs), vessels and cardiomyocytes. Some stromal subsets have been implied to interact with MPs and NTs through paracrine signaling. Epi, Epicardium; Sub epi, Subepicardium; Myo, Myocardium.

The ECM is a cell-free three-dimensional scaffold secreted by cells that provides structural integrity and biochemical and biomechanical signaling cues to surrounding cells (133). Epicardial-derived fibroblasts are an important source of ECM producing cells in the adult heart (14). Interestingly, the adult epicardium itself is also encased by ECM components which are lost after MI and subsequently re-formed (134). Not only does the epicardium rebuild its own ECM components, but also that of the regenerating heart. In newt, an organism that has comparable regenerative potential to zebrafish, resection injury induced epicardial enrichment of tenascin C (TSC), fibronectin (FN) and hyaluronic acid (HA) preceding the migration of progenitor cells, suggesting that the matrix directs progenitor cells toward the wound site (135). Similarly, in a zebrafish regeneration model it was found that FN is induced in the epicardium after cardiac damage. One of its receptors, *itgb3*, is upgregulated on cardiomyocytes near the injury site (25). Initially, *fn1* is expressed in the entire heart before becoming expressed in the epicardium near the injury site. Loss of FN expression disrupted cardiac regeneration, indicating that FN is required for this process in the zebrafish heart (25). Another study in zebrafish indicated a potential role for HA in cardiac regeneration. HA and its receptor hyaluronan-mediated motility receptor (Hmmr) were found to be essential for epicardial EMT and for migration of EPDCs into the ventricle. In rats, in the first few days after damage, both HA and HMMR were induced in the infarct area, indicating that this pathway may also be involved in cardiac repair in mammals (136). Concordantly, in embryonic mouse epicardial cells, TGFβ2 induced the production of HA and was partially required for the induction of epicardial cell differentiation and invasion *in vitro* (137), indicating a recapitulation of embryonic gene programs during injury on the level of ECM components. In a cryoinjury model in zebrafish, the ECM component collagen XII (ColXII) was found to be induced in the epicardial layer. Interestingly, ColXII in the epicardium and in fibrotic tissue had a heterogeneous cell source, being the epicardium, EPDCs, and cardiac fibroblasts. Additionally, the authors described that ColXII partially co-localizes with TSC and FN, two ECM components that were previously implicated in cardiac regeneration. The authors hypothesized that TGFβ signaling coordinates formation of a transient collagen network which contributes to an ECM conductive to cardiac regeneration (138). Recently, other ECM factors have been identified that may play a role in the cell-cycle of cardiomyocytes. Mass-spectrometry on the ECM of embryonic and postnatal hearts revealed that embryonic cardiac fibroblasts, which are derived from the epicardium, secrete SLIT2 and nephronectin (NPNT). Injections of these ECM proteins *in vivo* in postnatal mouse hearts promoted cardiomyocyte cytokinesis, indicating that the ECM composition could play an essential role in cardiac regeneration by inducing proliferation of cardiomyocytes (139). Agrin, a neonatal ECM protein found to regulate epicardial EMT during development (140), was shown to promote cardiac regeneration after MI *in vivo* in adult mice (141). Intriguingly, a single injection of recombinant human agrin into the pig hearts after MI was sufficient to improve cardiac function, which could be the result of cardioprotection, enhanced vascularization and cardiomyocyte proliferation (142). Overall, these studies show that components of the ECM, especially those produced by the epicardium or by EPDCs, can provide a target for inducing cardiac regeneration (Figure 2).

### Immunomodulation by the Adult Epicardium

The initial immune response after MI is mainly triggered by signaling from necrotic cells and is aimed at removing cell debris, ECM and dead cells. Subsequently, this inflammatory phase is repressed and followed by a reparative phase that allows for the deposition of ECM and the formation of a fibrotic scar that maintains integrity of the ventricular myocardium (143). The importance of a precisely regulated spatiotemporal response after MI is highlighted by reports that chronic unresolved inflammation enhances fibrosis and has a negative effect on function (143). The epicardium is involved in regulating the immune response through various routes. Already during development, hematopoietic cells (CD45^+^) cells are recruited to the epicardium that are distinct from Wt1^+^ cells, and after MI these cells are activated and migrate into the subepicardial space (134). During development, *Wt1* is required for the recruitment of epicardial macrophages (144) and in zebrafish, *wt1b* is expressed in a regenerative subset of macrophages after cryoinjury (145). Surprisingly, macrophages also contribute collagen to scar formation during heart regeneration in zebrafish and during cardiac repair in mouse, which was considered to be derived primarily from myofibroblasts (146). Inhibition of C/EBP mediated activation of *Wt1* and *Raldh2* after MI caused a significant reduction in neutrophil count and resulted in improved function, indicating a role for C/EBP mediated epicardial activation and subsequent leukocyte recruitment and inflammatory processes (105). Wt1 is reactivated in epicardial cells after injury (100), and yes-associated protein (YAP) and WW domain–containing transcription regulator 1 (TAZ) expression in Wt1^+^ cells is an important immunomodulator after injury (24). After MI, mice with a Wt1^CreERT2/+^ mediated deletion of YAP/TAZ had reduced expression of interferon-γ leading to impaired regulatory T-cell (Treg) recruitment to the myocardium, causing increased fibrosis, cardiomyopathy and death (24). In a zebrafish regeneration model, prostaglandin E_2_ (PGE_2_) and its receptor *ptger2* were shown to be upregulated after injury. COX enzymes catalyze the rate limiting step in the syntheses of prostanoids such as PGE_2_, and epicardial cells were observed to have a higher expression of *cox2a* in their model compared to macrophages and other cardiac cell types (147). Additionally, small molecule inhibitors of Cox2 activity led to decreased PGE_2_ concentrations and cardiomyocyte proliferation, indicating that Cox2 drives PGE_2_ synthesis and CM proliferation during heart regeneration (147). Interestingly, PGE_2_ has also been associated with YAP activation and Treg recruitment, indicating a potential interaction within the epicardium that modulates the inflammatory response (147). In conclusion, the epicardium is an important mediator of the inflammatory response after MI (Figure 2), which can potentially be modulated to improve cardiac repair.

## The Composition of the Epicardium

As we have described, the adult epicardium recapitulates several of its embryonic capacities, such as proliferation, EMT, and migration to contribute to cardiac repair. However, in the absence of external stimuli like Tβ4, these processes appear to be less efficient in the adult epicardium compared to its developing counterpart. A potential difference in capacity could derive from differences in the cellular composition of the epicardium in development or in the adult heart. It remains unknown if epicardial cells have a uniform function during development and after injury, or if subsets of cells exist within the layer that contribute more to cardiac development and repair. Therefore, it is vital to know the composition of the epicardium during development and in the adult heart in order to optimize the post-injury response, below we will address the most recent knowledge.

### Cellular Heterogeneity in the Developing Epicardium

Since epicardial cells have the potential to differentiate into various cardiac cell types, it is hypothesized that the epicardial layer is not composed of one specific cell type. This concept was supported by the notion that the source of the epicardium, the PEO, is a heterogeneous cell cluster consisting of endothelial cells (ECs) (148) within a mesenchymal core, covered by an epithelial outer layer, which can all be characterized by the expression of specific markers or combinations thereof. Analysis of the ECs in the PEO revealed that these cells have a heterogeneous origin from the PEO itself, the liver bud and the sinus venosus. The ECs in the PEO are immature and likely provide nutritional support (148, 149). However, there has been no indication that these ECs contribute to the developing heart (149). The previously mentioned observation that well-established epicardial markers Wt1, Tcf21, and Tbx18 are heterogeneously expressed in both the PEO and the developing chick and mouse epicardium (35, 57) supports the hypothesis of a heterogenous epicardium. However, through lineage tracing experiments in mice it was shown that proepicardial cells from the mesenchymal core expressing SEMA3D or Scx may comprise a proepicardial subcompartment that specifically contributes to the formation of the coronary vasculature (35). In contrast, a recent study showed that the expression of *Sema3d* and *Scx* overlaps with other epicardial markers in the PEO. Exclusive expression of *Sema3d* and *Scx* was observed in the septum transversum, but these cells did not appear to contribute to the developing heart (59). Unfortunately, the ability to definitively study cell fate of EPDCs has thus far been limited due to the non-uniform expression of transcription factors in epicardium, and to the use of Cre-based lineage tracing models that are either not sufficiently specific to the epicardium or that fail to label all cells within the entire layer. However, the recent advent of single cell RNA sequencing, with which the transcriptome of individual cells can be identified, has greatly simplified the identification and composition of cell populations within a tissue, as well as their differentiation trajectories. Another suggestion of proepicardial heterogeneity was established by Tyser et al. A novel source of proepicardial cells was identified dubbed the juxta-cardiac field (JCF), through a combination of single-cell RNA sequencing and genetic lineage tracing from early cardiac development (E7.5) onwards. When tracing the fate of these JCF-derived cells, they were found to contribute to both the PEO and subsequently to the epicardium and/or to cardiomyocytes in the developing mouse heart (150). These data suggest that there are populations contributing to the PEO that may have specific abilities to differentiate into various other cell types besides ECs. The question remains whether or not these cells can differentiate into both cell types, generating epicardial cells that can continue to become cardiomyocytes or if they are bipotent cells from the onset that can become either epicardial cells or cardiomyocytes (150). When looking beyond the PEO, in the developing epicardium there are also several indications supporting the hypothesis of heterogeneity. The human ventricular epicardium has been described to be formed as a multiple-cell layered epicardium while the atria have a single-cell layer epicardium, suggesting that localization of epicardial cells could influence their behavior (34). Concordantly, when unraveling the role of ECM components in the developing mouse heart, a morphologically heterogeneous epicardium was observed related to the EMT-status of epicardial cells. Epicardial cells undergoing EMT were located near regions with a distinct ECM composition, composed of less integrin α4 and laminin and more agrin puncta. Conversely, loss of agrin resulted in fewer Wt1-positive cells in the epicardium and the myocardium and an increase in β-catenin, suggesting more cell-cell adhesion and thereby a decreased ability to undergo EMT (140). These data suggest that epicardial differentiation is affected not only by the transcriptome and secretome of epicardial cells but also by the ECM that is formed by epicardial cells and its derivatives during cardiac development. Interestingly, ECM components were also found to influence epicardial EMT and were upregulated in EPDCs during EMT and migration (118). Additionally, bone-marrow derived CD45^+^ cells were found within the epicardium, indicating that there are other cell populations besides the PEO that contribute to the cellularity of the epicardial layer (134). Various studies have recently tried to deconvolute the composition of the developing epicardium using scRNA seq. Weinberger et al. identified three functional subpopulations within the developing zebrafish epicardium at 5 days post-fertilization (5 dpf) by sequencing cells from reporter fish lines for *tcf21, wt1b*, and *tbx18*. Analysis of transcriptomes of these cells showed three distinct epicardial clusters. The function of these subpopulations was confirmed using newly generated knockout zebrafish for the markers found therein. One of the subpopulations expressed *transglutaminase 2b* (*tgm2b*), and both transient and stable somatic knockdown of this gene led to defects in the epicardial layer. This suggests *tgm2b* plays a crucial role in maintaining the integrity of the epicardial sheet during its formation. By creating somatic knock-out animals for genes found in the other subpopulations, the authors observed that *sema3fb* and *cxcl12a* had distinct effects on epicardial migration and composition, respectively. *Sema3fb* was strongly expressed within the bulbous arteriosus (BA), a part of the outflow tract. *Sema3fb* knockout regulated the number of *tbx18*-positive cells contributing to SMCs covering the outflow tract. The third population, which was enriched for *cxcl12a*, was spatially restricted to an area between the BA and atrium. Knockout of this gene revealed that this cell population was involved in homing of leukocytes to the developing heart (151), establishing a mechanism for the contribution of CD45+ cells of non-PEO origin to the developing epicardium (134). The finding of these three subpopulations suggests that the epicardium could be heterogenous in zebrafish during development, and that these epicardial subpopulations are spatially and functionally distinct. A potential regulator of epicardial heterogeneity has been identified by a scRNA seq study on epicardial cells derived from human pluripotent stem cells. Gambardella et al. found that basonuclin (BNC1) can modulate the expression of essential epicardial transcription factors Wt1 and Tcf21. In the absence of BNC1 cells have an increased expression of Tcf21 and a reduced expression of Wt1 (152), indicating that BNC1 can influence the phenotype of epicardial cells. It is important to note that these two studies described above were performed in different organisms and models and are confined to a limited developmental timeframe. Additionally, it is not clear whether these cells are veritable epicardial cells that are located on the outside of the heart, or EPDCs that are undergoing EMT and initiated differentiation (59). Also, studies using cell culture models could have a bias toward certain cell states due to the culture conditions, a lack of interactions with surrounding tissues and a proper developmental progression. Therefore, more evidence on the epicardial cellular composition based on other models is still necessary.

Another source of information regarding the epicardial heterogenicity could derive from cardiac cell atlases that have been generated using scRNA seq to identify rare cell populations and interactions within the developing heart. In these studies, the epicardium is often annotated but its potential heterogeneity is often overlooked due to low epicardial cells numbers relative to the total number of cardiac cells, or heterogeneity is ascribed to developmental progression (153–156). Another possible explanation is that in these studies epicardial cells are characterized based on known markers and not further scrutinized, thus novel (sub)populations are potentially not identified.

New insights regarding the composition of the developing epicardium have come from several studies focusing on spatiotemporal analysis. Contrary to previous studies (151, 152), a larger developmental timeframe is studied by performing sequencing at several timepoints. Liu et al. used scRNA seq at three developmental stages (early to late septation) of the developing outflow tract in mice, a transient structure that gives rise to the aorta and the pulmonary trunk. Although they revealed two epicardial populations that were heterogenous in their composition, this was most likely the result from developmental progression, rather than different subpopulations (157). In the human heart, a combination of spatial transcriptomics and scRNA seq at various timepoints during development [4.5–9 weeks post-conception (PCW)] was able to identify the epicardium in all stages. In this dataset there appeared to be no heterogeneity in the epicardial cells, but mesenchymal cells showed heterogeneity based on their expression of marker genes. However, due to the low number of cells sequenced (3,717 in total) at the intermediate stage (6.5–7 PCW), the low resolution of their spatial transcriptomic approach (~30 cells per spot), and the limited number of genes used in validation through *in situ sequencing*, it is difficult to draw a concrete conclusion about epicardial composition (158). In a similar study in which spatiotemporal analysis of the heart was performed during key developmental stages in chicken, 5,621 epicardial cells from the ventricular free wall were clustered. In their analysis, the epicardial cells and its derivatives (EPDCs) clustered based on their position within the differentiation process. The data were able to confirm that epicardial cells follow the anticipated trajectory where they undergo EMT and migrate into the myocardium before committing to either fibroblast or mural cell fate (118). Although no functional epicardial heterogeneity was observed, cells that have started EMT might reside in the epicardial layer during later stages of development (day 7) and may continue to stay in the epicardium in an undifferentiated intermediate phenotype until day 10. This variation in differentiation state could give the impression of heterogeneity in the epicardial layer if observed at a singular timepoint, but these data suggest that this heterogeneity does not stem from a difference in initial cell population. The finding that the epicardium lacks functional heterogeneity was corroborated in a study where the developmental stages from the formation of the PEO (E9.5), the establishment of the epicardium (E13.5) up until the differentiation of EPDCs (E15.5) were investigated. Here, established markers such as *Wt1, Tbx18, Tcf21, Scx*, and *Sema3d* did not demarcate functional subpopulations. Moreover, they showed that expression of these markers did not influence the differentiation trajectory of EPDCs to either mural cells or fibroblasts, and that EPDCs lost expression of these markers upon the induction of EMT (with the exception of *Tcf21*, which goes up after EMT until differentiation) (59). This further illustrates that subpopulations (see Table 1) are more likely to be a result of developmental progression and that reported heterogeneity is rather a reflection of transcriptional changes after EMT. Additionally, it has been suggested that the various differentiation trajectories of EPDCs might be due to extrinsic cues such as paracrine factors and location relative to cardiac cell types (e.g., ECs) and ECM rather than intrinsic expression of transcription factors (59).

**Table 1:** Overview of findings in search of heterogeneity in developing epicardium.

**Model**	**Technique**	**Timepoint**	**Sequenced tissue**	**Finding**	**References**
Zebrafish reporter/knockout lines	scRNA seq, hybridization chain reaction	5 dpf	*Wt1b* (47 cells), *tcf21* (137 cells)*, tbx18* (52 cells) from reporter lines	Distinct functions for *tgm2b, cxcl12a, sema3fb* in epicardial development	(151)
hPSC-epicardium	scRNA seq/BNC1 knockdown	-	232 hPSC-epi single cells	BNC1 drives heterogeneity	(152)
Human embryonic hearts	Spatial transcriptomics (ST), scRNA seq, *in situ* sequencing, smFISH	4.5–9 PCW	ST: 3115 spots containing ~30 cells. scRNA seq: 3717 cells from 6.5 to 7 PCW	Epicardial cells displayed no heterogeneity. Heterogeneity was observed in mesenchymal cells	(158)
Chicken embryonic hearts	Spatial transcriptomics (ST), scRNA seq, smFISH	4–14 days (HH21–HH40)	ST: 6,800 barcoded spots (10–20 cells per spot). scRNA seq: 22,315 cells	No functional heterogeneity: post-EMT cells residing in epicardium	(118)
Wt1^CreERT2^;Rosa26^tdTom^	ISH, scRNA seq	E9.25–E15.5	Published datasets (E9.25–E10.5) - 276 tdTom+ cells at E15.5	Epicardial cells displayed no heterogeneity. Heterogeneity was observed in mesenchymal cells at E15.5	(59)

### Heterogeneity in the Postnatal Epicardium

The composition of the fetal epicardium, although subject to debate, has been researched intensively (see section Cellular heterogeneity in the developing epicardium and Table 1), but very little is known about the composition of the epicardium in the adult heart. In its quiescent state, very few cells express markers such as Wt1 that denote activated epicardium and its functional heterogeneity is likely limited. However, since ischemic injury induces re-activation of the epicardial layer, identifying subpopulations that participate in the wound healing process either through cellular contributions, or *via* paracrine signaling, could result in the identification of mechanisms that aid in cardiac repair. A mouse model of cardiac ischemia/reperfusion (I/R) injury revealed that after 7 days Wt1, Tcf21, and Tbx18 were expressed in distinct as well as in overlapping populations within the subepicardial mesenchyme (102). In the same study, reactivation of Tcf21 and Wt1 was found in interstitial fibroblasts and not myofibroblasts after I/R, indicating that these markers are present in differentiated fibroblasts, but not activated myofibroblasts. This suggests that the expression of these markers coincides with the induction of fibrogenesis after I/R (102). To establish whether injury mediated activation of the epicardium results in similar effects as during development, a direct comparison between embryonic EPDCs (E12.5) and adult EDPCs after Tß4 priming and MI (2, 4, and 7 days post-MI) was performed. A majority of Wt1-positive cells in the adult cells expressed stem-cell antigen-1 (Sca-1) compared to their embryonic counterpart (159). Sca-1^+^ cells are considered a progenitor cell population for various cell populations, such as CMs, ECs, SMC and fibroblasts, although their adoption of CM fate is debated (160, 161). Wt1^+^Sca-1^+^ displayed increased expression of mesenchymal markers CD105, CD44, Thy-1, and PDGFRb compared to embryonic EPDCs, and a heterogenous expression of these markers (159). Although this reactivation is not autonomous, i.e., it is stimulated by Tß4, it does indicate that subpopulations in the activated adult epicardium may have distinct functions. Interestingly, a similar degree of heterogeneity was observed in epicardial cells 5 days post-MI without external activation (162). However, from these studies it is unclear if the different populations arose from a common ancestor cell in the epicardium or if heterogeneity pre-existed within the inactive epicardial layer.

Using scRNA seq in *tcf21*^+^ epicardial cells of adult zebrafish, Cao et al. report three subpopulations with a distinct gene expression signature in the uninjured heart and found that this heterogeneity persisted after injury (163). In this study, only a few dozen cells were sequenced which may make the interpretation more challenging. When comparing transcriptional changes between a model for mitochondrial cardiomyopathy and healthy postnatal mouse hearts the epicardium displayed no heterogeneity, although this may be due to the disease model used (164). Recently, the epicardial layer and subepicardial mesenchyme (or epicardial stromal cells – epiSCs) were subjected to single cell sequencing 5 days post-MI by Hesse et al. (119). This approach provided a higher resolution by focusing on the epicardial layer specifically. Interestingly, they described heterogeneity in the epicardial layer which was partly due to a proliferative phenotype and a high degree of protein synthesis. The function of the identified subpopulations was not assessed in mice, nor whether these populations resulted from differential progression, as observed during development. Nevertheless, a high degree of heterogeneity was detected in stromal and ECM producing cells (119) (see below). Strikingly, there was some conservation between these data and functionally heterogenous epicardial subpopulations in zebrafish as described by Weinberger et al. (119, 151). Since a tamoxifen-inducible Wt1 reporter line was used to obtain the scRNA seq data, the contribution of Wt1^+^-derived cells to the identified populations could be analyzed. In this set-up no contribution of traced cells to Wt1^−^ populations was observed within the 5 days post-MI (119). In a subset of the Wt1^+^ population there was a high expression of *Tmbs4x*, the gene coding for Tß4 which can induce cardiomyogenesis *in vivo* and induce cardiac repair when given prior to injury (99, 120). A similar cellular subset has been reported in the chicken epicardium during development (118). Based on their data, Hesse et al. also hypothesized that a subset of the Wt1^+^ cells may have cardiomyogenic potential due to their high expression of cardiac specification markers and sarcomere proteins. Additionally, they found that all stromal cells expressed paracrine factors previously observed in Wt1^+^ cells, and that this was not exclusive to epicardial stromal cells but also in myocardial stromal cells (119). Although this study investigates the epicardial composition post-MI in a high resolution, it does not address the function and differentiation trajectory of observed subpopulations. Since only a singular timepoint is analyzed (5 days post-MI), a conclusion whether or not these subpopulations are a reflection of functional differences or of a varying differentiation state cannot be made. In a study where scar formation was analyzed over multiple timepoints, a similar degree of heterogeneity was observed in stromal cells compared to the study by Hesse et al. Using Wt1^Cre^;RosaZsGreen^f/+^ mice to label epicardial derivates at day 0 (d0), d1, d3-5,7, d14, and d28, the evolution of mesenchymal cells was identified. A novel subpopulation was identified dubbed injury response (IR) cells at d1. The IR subpopulation had a high expression of monocyte-macrophage chemoattractants *Ccl2, Ccl7*, and *Csf1* and neutrophil activators *Cxcl1* and *Cxcl5*. Additionally, it also expressed pro-inflammatory and pro-fibrotic factors *Il33, Cxcl12*, and *Tgfb12*. These IR cells transitioned to myofibroblasts at d3, and myofibroblasts also displayed heterogeneity. A subpopulation of myofibroblasts was similar to recently identified matrifibrocytes (MFCs) in the mature scar at d14-d28. MFCs were identified as cells that express high levels of ECM genes and support the integrity of the mature scar in injured hearts (165). At this stage, late-resolution (LR) fibroblasts were also found, expressing genes associated with differentiation and regulation of matrix remodeling and deposition (166). In general, while more information regarding the adult epicardium and its composition is becoming available, the current studies indicate the need for further analysis at multiple timepoints of mesenchymal subpopulations and their function in cardiac repair and regeneration. The epicardial layer seems to lack functional heterogeneity in the adult heart after injury, but scRNA seq analysis could shed more light on the role of paracrine factors and cellular contributions of the epicardium and its derivatives post-injury (Figure 2).

## Conclusions

The epicardium has been unequivocally shown to be essential during cardiac development, both *via* the contribution of cells and through the secretion of paracrine factors. Since these processes are also required to repair the heart after injury, the epicardium has been considered a very appealing source for endogenous cardiac repair. It has become clear that most processes that occur within the epicardial setting during development are recapitulated in the adult epicardium after injury, albeit less efficient. Therefore, there has been an interest in deconvoluting the epicardial composition to identify targets to optimize the post-injury response. High-resolution analysis of the epicardial layer in the developing heart has suggested heterogeneity within the layer. However, this is mostly the case in studies where a limited timeframe has been studied. In more elaborate approaches where development through time has been investigated, the heterogeneity seems to be explained by developmental progression, i.e., cells are in different stages of EMT or are in the process of differentiating into a specific cell type. There is very limited evidence that the heterogeneity derives from distinct subsets of cells. This is also true for the PEO; while there is a certain degree of heterogeneity this does not account for the different cell types, apart from perhaps cells from the JCF. In the adult heart, the epicardium is in an inactive state and likely has very little heterogeneity, but analysis of the epicardium after injury has suggested he presence of subpopulations in epicardial-derived mesenchymal cells during cardiac repair. Overall, our knowledge on the composition of this intriguing cell population is steadily increasing through the advent of novel techniques such as single RNA seq. Moreover, besides knowledge on its cellular composition, a vast amount of data has been accrued regarding novel proteins, signaling pathways or paracrine factors produced by epicardial cells in regenerative and non-regenerative species. Therefore, whether or not a specific population can be identified and targeted to stimulate repair, other approaches such as delivery of epicardium related paracrine factors, specific modulation of the ECM or the immune response can provide additional means to further enhance the development of novel therapies to repair the injured heart.

## Author Contributions

TS and AS contributed to the conception and design of the manuscript. TS wrote the first draft of the manuscript. AS wrote sections of the manuscript. Both authors contributed to manuscript revision, read, and approved the submitted version.

## Funding

This work was supported by the Dutch Heart Foundation (Senior Dekker Grant 2017T059 to AS).

## Conflict of Interest

The authors declare that the research was conducted in the absence of any commercial or financial relationships that could be construed as a potential conflict of interest.

## Publisher's Note

All claims expressed in this article are solely those of the authors and do not necessarily represent those of their affiliated organizations, or those of the publisher, the editors and the reviewers. Any product that may be evaluated in this article, or claim that may be made by its manufacturer, is not guaranteed or endorsed by the publisher.
